# Update of the FANTOM web resource: expansion to provide additional transcriptome atlases

**DOI:** 10.1093/nar/gky1099

**Published:** 2018-11-08

**Authors:** Marina Lizio, Imad Abugessaisa, Shuhei Noguchi, Atsushi Kondo, Akira Hasegawa, Chung Chau Hon, Michiel de Hoon, Jessica Severin, Shinya Oki, Yoshihide Hayashizaki, Piero Carninci, Takeya Kasukawa, Hideya Kawaji

**Affiliations:** 1 RIKEN Center for Integrative Medical Sciences, Yokohama, Kanagawa 230-0045, Japan; 2 Department of Developmental Biology, Graduate School of Medical Sciences, Kyushu University, Fukuoka 812-8582, Japan; 3 RIKEN Preventive Medicine and Diagnosis Innovation Program, Wako, Saitama 351-0198 Japan

## Abstract

The FANTOM web resource (http://fantom.gsc.riken.jp/) was developed to provide easy access to the data produced by the FANTOM project. It contains the most complete and comprehensive sets of actively transcribed enhancers and promoters in the human and mouse genomes. We determined the transcription activities of these regulatory elements by CAGE (Cap Analysis of Gene Expression) for both steady and dynamic cellular states in all major and some rare cell types, consecutive stages of differentiation and responses to stimuli. We have expanded the resource by employing different assays, such as RNA-seq, short RNA-seq and a paired-end protocol for CAGE (CAGEscan), to provide new angles to study the transcriptome. That yielded additional atlases of long noncoding RNAs, miRNAs and their promoters. We have also expanded the CAGE analysis to cover rat, dog, chicken, and macaque species for a limited number of cell types. The CAGE data obtained from human and mouse were reprocessed to make them available on the latest genome assemblies. Here, we report the recent updates of both data and interfaces in the FANTOM web resource.

## INTRODUCTION

Transcription is the first process in the synthesis of molecules that carry out functions encoded on the genome. Promoters and enhancers are key classes of *cis*-regulatory elements controlling the transcription of target genes from proximal upstream regions and distal regions, respectively. Elements of both of these classes are transcribed (1).

FANTOM (functional annotation of mammalian genomes) is an international collaborative effort that aims to study genomes from the perspective of the transcriptome (2–6). Its fifth iteration (FANTOM5) drafted atlases of promoters and enhancers and profiled dynamic changes in their activities during differentiation and responses to stimuli (4,7,8). These studies were based on transcription initiation profiles at nucleotide resolution detected using CAGE (cap analysis of gene expression) (9), the most accurate experimental approach to identify 5′-ends of capped RNAs (10). c*is*-regulatory elements were monitored in more than 1800 samples from human and 1000 samples from mouse (11), then the resulting data were compiled and made available via the FANTOM web resource (12,13).

**Figure 1.**
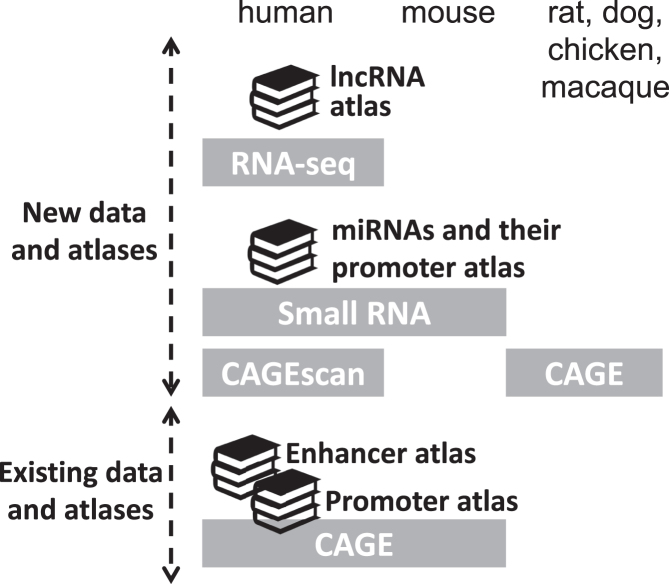
Schematic view of the FANTOM5 atlases and data. The atlases are indicated by book icons, and the data sets are indicated by gray boxes. New data sets and atlases are indicated in the top panel.

The FANTOM5 project recently provided atlases of long noncoding RNAs and micro RNAs and their promoters, with accompanying RNA-seq and short RNA transcriptome data (Figure 1). The project also provided additional transcriptome data, such as CAGE profiles from rhesus macaque (*Macaca mulatta*), chicken (*Gallus gallus*), dog (*Canis lupus familiaris*), and rat (*Rattus norvegicus*) and CAGEscan (14) profiles that can associate promoters with their cognate exons with paired-end sequencing (Figure 1). Here, we introduce the recent updates of the FANTOM web resource.

## RESULTS

### Long noncoding RNA atlas with matched RNA-seq

**Figure 2.**
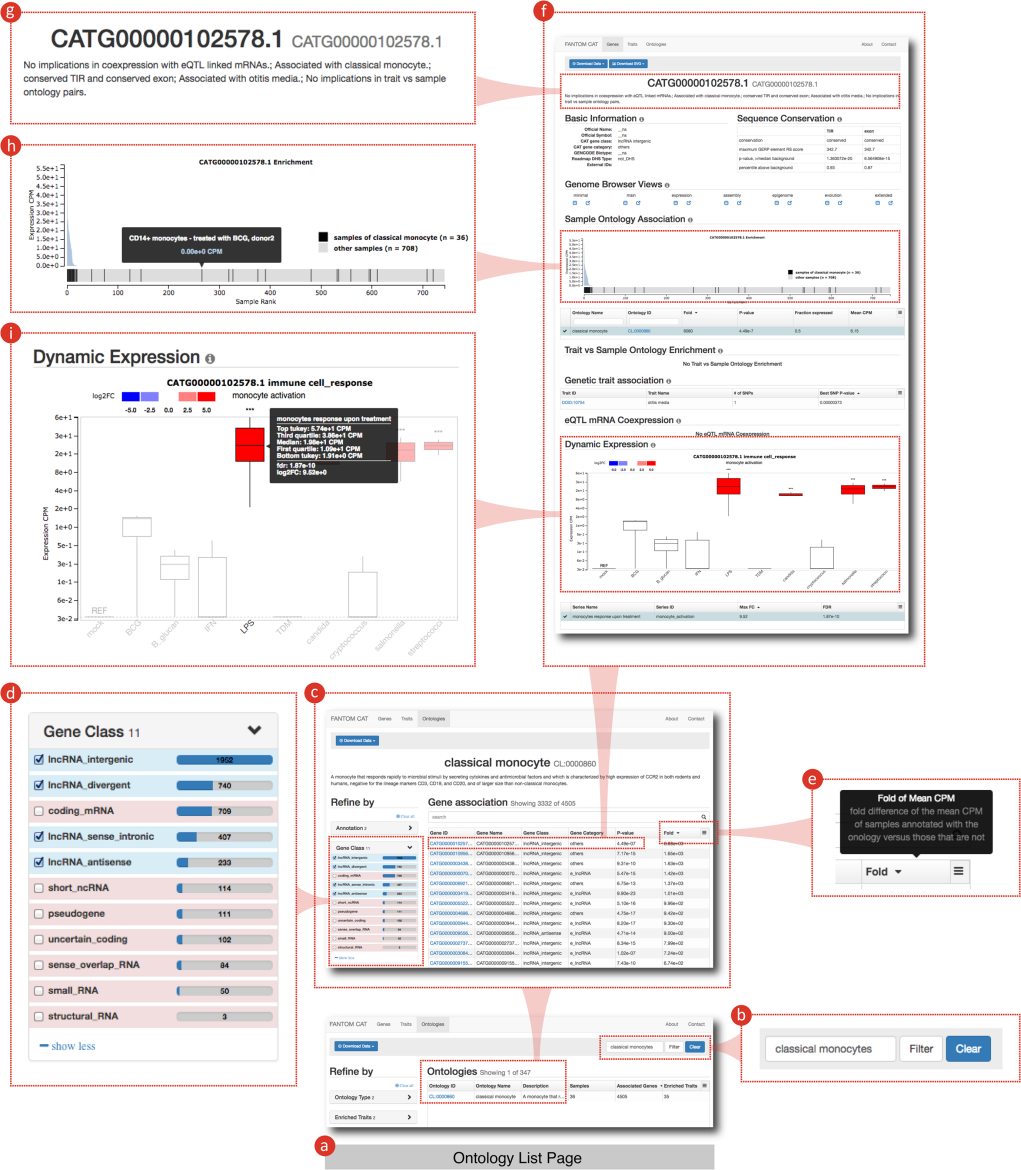
Using the FANTOM CAT browser to explore lncRNAs associated with classical monocytes. (**A**) Browse the ontology list page by clicking on ‘Ontologies’ on the landing page; (**B**) filter for ‘classical monocytes’; (**C**) list of genes associated with ontology CL:0000860 classical monocytes; (**D**) filter for gene classes of ‘lncRNA’; (**E**) sort genes by their level of association with classical monocytes; (**F**) summary page of the lncRNA gene CATG00000102578.1, with highest level of association to classical monocytes (http://fantom.gsc.riken.jp/cat/v1/#/genes/CATG00000102578.1); (**G**) annotation summary of CATG00000102578.1; (**H**) expression level of CATG00000102578.1 in FANTOM5 samples, with samples of classical monocytes highlighted; (**I**) dynamic expression pattern of CATG00000102578.1 in FANTOM5 samples.

The human genome is pervasively transcribed and produces a large number of long noncoding RNAs (lncRNAs) (15,16). However, the current annotation of lncRNAs is far from complete (17). We attempted to comprehensively annotate human lncRNAs by integrating CAGE and RNA-seq on a large scale. We performed RNA-seq on a subset of FANTOM5 human samples (*n* = 70) and assessed the compatibility of the resulting transcriptome assembly with CAGE-defined transcription start sites (TSSs). Based on the matched CAGE and RNA-seq datasets, we formulated a custom metric, the transcription initiation evidence score (TIEScore), to quantitatively integrate CAGE-defined TSSs and transcriptome assemblies (18). We then applied the TIEScore to our own RNA-seq assembly and to other transcriptome assemblies and gene models (Human Body Map 2.0 (19), miTranscriptome (20), and GENCODE (21)), to build a meta-assembly of transcripts with accurate 5′-ends, called FANTOM CAT (CAGE-Associated Transcriptome). At a robust TIEScore cutoff, FANTOM CAT consists of 59 110 genes, including 27 919 lncRNAs (18). All genes in FANTOM CAT were classified based on their genomic (e.g. intergenic or antisense) and epigenomic (e.g. promoter- or enhancer-derived) contexts. They were then annotated with associations to genetic traits (using known GWAS loci) and to sample ontologies (using FANTOM5 expression profiles and sample ontologies). In addition, their dynamic expression patterns among FANTOM5 samples were annotated based on differential expression analyses. Finally, we integrated the GTEx expression quantitative trait loci (eQTL) data (22) with the FANTOM5 expression profiles to define a set of lncRNAs with potential roles in transcriptional regulation. All these annotations, as well as the FANTOM CAT meta-assemblies at various TIEScore cutoffs, are available at http://fantom.gsc.riken.jp/cat. The FANTOM CAT browser (Figure 2) allows interactive visualization of genes and their annotations (http://fantom.gsc.riken.jp/cat/v1/#/). The raw reads and alignments are available from the FANTOM5 data files repository (http://fantom.gsc.riken.jp/5/datafiles/phase2.5/).

### Atlas of miRNAs and their promoters with matched short RNA-seq

**Figure 3.**
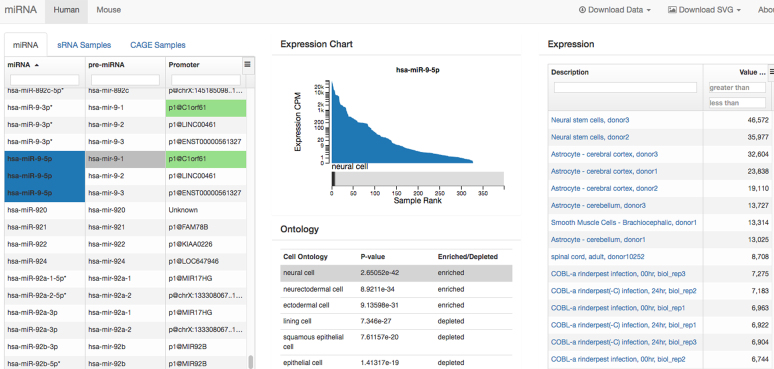
The FANTOM5 miRNA expression atlas. The annotated miRNA promoter, expression profile and results of the cell-type enrichment analysis are shown by using miRNA hsa-miR-9-5p as an example (http://fantom.gsc.riken.jp/5/suppl/De_Rie_et_al_2017/vis_viewer/#/human).

In addition to the synthesis of RNA during transcription, gene expression also depends on post-transcriptional processes such as RNA stabilization, translation efficiency, and degradation. Micro RNAs (miRNAs) are a major class of small noncoding RNAs involved in post-transcriptional regulation by repressing target genes via RNA degradation or inhibition of translation. As part of FANTOM5, we created an atlas of miRNA expression by sequencing short RNA libraries produced from a subset of the human and mouse RNA samples that were used for the promoter atlas (23). The atlas comprises 422 libraries of human samples and 78 libraries of mouse samples; most samples were derived from human primary cells. Expression levels of all annotated miRNAs, as well as candidate novel miRNAs discovered using miRDeep2 (24), were calculated for each sample. The enrichment or depletion of particular miRNAs in specific human primary cell types was then evaluated (Figure 3). Promoters were systematically identified for human and mouse miRNAs, using the FANTOM5 CAGE data to identify transcription start sites at the single-nucleotide resolution. Expression levels of mature miRNAs, as measured by short RNA library sequencing, were correlated with the expression level of the corresponding primary miRNA transcript in the matching CAGE library. Thus we can use the CAGE expression signal at the miRNA promoter as a proxy for the expression level of the mature miRNA, and to extend the miRNA expression atlas to all CAGE samples in FANTOM5. The FANTOM5 miRNA atlas with the annotated promoter for each miRNA, the expression profiles of the mature and primary miRNAs, and the cell-type enrichment analysis is available at http://fantom.gsc.riken.jp/5/suppl/De_Rie_et_al_2017/ (Figure 3). A clickable heatmap of miRNA expression levels in human primary cell samples is available at http://fantom.gsc.riken.jp/5/suppl/De_Rie_et_al_2017/vis_viewer/#/heatmap. The raw reads and alignments are available as part of the FANTOM5 data files (http://fantom.gsc.riken.jp/5/datafiles/phase2.5/).

### CAGEscan to link orphan promoters to transcriptional unit

While the application of CAGE to ∼1800 human samples enabled us to identify nearly 200 000 promoter regions, only half of them were associated with existing gene models (4) and characterizing the rest remains a challenge. One approach to improve genome annotation is to perform RNA-seq from the matched samples, as described above; however, the efficiency of this method in capturing the terminal ends of transcripts is limited. CAGEscan is an experimental protocol that links transcription initiation sites to cognate exons by paired-end sequencing of random-primed sites of cDNAs (14). We applied CAGEscan to a subset of human samples (*n* = 56) from the promoter atlas (25). Aligned pairs starting in the same region were assembled into transcript models called CAGEscan clusters and added to the FANTOM5 data files together with the raw data (http://fantom.gsc.riken.jp/5/datafiles/phase2.5/).

### Complementary CAGE profiles for macaque, chicken, rat and dog

To complement the promoter atlases created for human and mouse, we obtained CAGE profiles from rhesus macaque, chicken, dog and rat. The macaque samples cover 15 brain regions that match the brain regions samples profiled in human (26). The chicken samples cover consecutive embryonic stages, providing snapshots of promoter activation and usage during development (27). Additional samples from chicken include adult primary mesenchymal stem cells, aortic smooth muscle cells, and hepatocytes. These cell types match the cell types in the other two species (rat and dog) (28), as well as in human and mouse. CAGE profiles from these four species were processed as performed for human and mouse, including the identification of CAGE peaks (4). Although the coverage of samples is far more limited than in human and mouse, our experimental design of matched samples, application of the same CAGE library preparation protocol, and identical data processing pipeline enables us to study transcriptional regulation across species. All the processed data are integrated into the FANTOM5 data files (http://fantom.gsc.riken.jp/5/datafiles/phase2.5/).

### Up-to-date promoter atlases based on GRCh38 and GRCm38

All of the transcriptome atlases discussed above were built based on GRCh37 for human and GRCm37 for mouse, which were the latest reference genome assemblies at the time of the launch of the FANTOM5 project. Since the improved GRCh38 and GRCm38 reference genome assemblies were released for human and mouse, thanks to extensive efforts by the Genome Reference Consortium (29), we updated the promoter atlases accordingly by realigning the CAGE reads to the new reference genomes and recalculating the TSS frequencies. We further converted the genomic coordinates of the CAGE peaks from GRCh37 to GRCh38 and from GRCm37 to GRCm38 via UCSC’s liftOver tool, and used the same identifiers to ensure compatibility between genome assemblies. We carefully assessed the results of the conversion and found that >99% of the CAGE peaks were successfully converted. A handful of regions with issues after liftOver were curated manually (30). Finally, the converted CAGE peaks were merged with those called *de novo* to remedy the absence of converted peaks in the latest genomes due to differences between the two assemblies. The annotations of the peaks, such as names and gene associations, were also updated based on the most recent gene models (30). The resulting data are available from the web resource at http://fantom.gsc.riken.jp/5/datafiles/reprocessed/.

### Update of the FANTOM5 interfaces

To increase accessibility to the datasets added, we loaded them into the FANTOM5 interfaces and databases (12,13). In addition to the expansion of data sets in the data archive, the ZENBU genome browser (31) now provides visualizations for the new data sets of CAGE and RNA-seq, as well as annotation data on the latest genome assemblies. The SSTAR browser (32) has been updated to include sample information and CAGE peaks for the dog, rat, macaque, and chicken samples. The sample information is of particular importance in this context, not only because of the matching of RNA sources across species, but also because the same RNAs were profiled by multiple protocols (CAGE, RNA-seq, short RNA-seq and CAGEscan). SSTAR provides a complete set of metadata to connect all of them based on common information. Semantic MediaWiki—the backend system of SSTAR—has been upgraded to improve performance. TET, the FANTOM5 table extraction tool, now provides CAGE peaks and expression levels for the four new species, enabling researchers to obtain subsets of data of their interest from the large data files.

**Figure 4.**
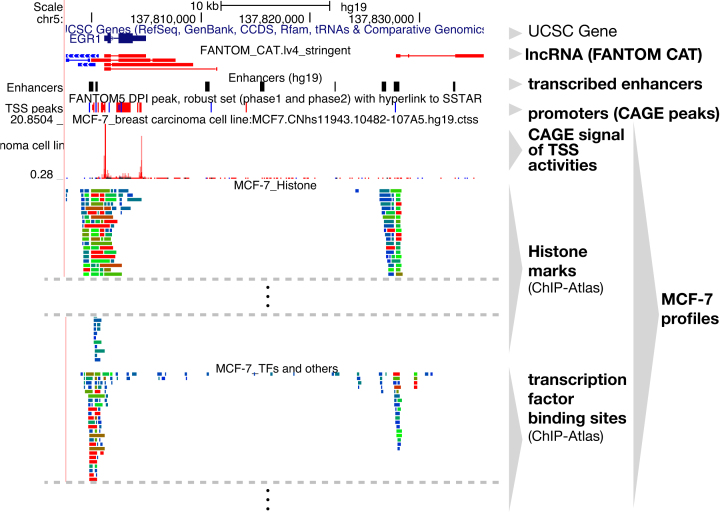
MCF-7 cells: integrated view of transcriptome and epigenetic marks. The data were obtained from ChIP-Atlas, which covers all ChIP-seq experiments deposited in SRA, in addition to FANTOM5. The view of EGR-1 locus is displayed by the UCSC Genome Browser via the trackHub framework(https://genome-asia.ucsc.edu/cgi-bin/hgTracks?db=hg19&hubUrl=http://fantom.gsc.riken.jp/5prim/external/CellLines-CAGE-with-epigenome/hub.txt).

We have updated the configuration of trackHub (33) to reflect the latest data, visible in both the UCSC Genome Browser (34) and the ENSEMBL Genome Browser (35). Because the epigenetic machinery plays crucial roles in controlling transcriptional regulation, we have further extended the contents of the FANTOM web resource by including epigenetic data from the ChIP-Atlas (Figure 4), a comprehensive database of public ChIP-seq and DNase-seq data available via the Sequence Read Archive (SRA). The data it contains have been uniformly reprocessed and annotated (36). We have manually curated epigenetics data of cell lines corresponding to those profiled by CAGE and made the data available via the trackHub configuration tool (bioRxiv: https://doi.org/10.1101/314807). ChIP-Atlas integration with transcription initiation activities will facilitate the study of transcriptional regulation. The collected data files with the configuration file (hub.txt) are accessible at http://fantom.gsc.riken.jp/5prim/external/CellLines-CAGE-with-epigenome/.

## DISCUSSION

With this latest update of the FANTOM web resource, we focused mainly on diversifying the types of assays rather than increasing the number of samples for the same assay because CAGE technology allows us to identify the 5′-ends of capped RNAs but not the internal structure of the RNAs. We sought to overcome this limitation by integrating data sets targeting different portions of the RNA, and thus to understand the transcriptome from a more comprehensive perspective. We expanded our resource to encompass lncRNAs and miRNAs, whose atlases further our understanding cellular regulatory mechanisms; however, the functional roles of these molecules—in particular lncRNAs—largely remain to be elucidated. The sixth iteration of FANTOM is thus aimed at specifically assessing lncRNA functions in multiple cell types (37).

We have also expanded the number of samples by covering four additional species—rat, dog, chicken and macaque — for a limited amount of cell types. As the comparison of multiple genome sequences enables us to study the evolutionary processes of genetic inheritance, cross-species transcriptome comparisons make it possible in turn to assess the functional consequences of evolution at the molecular level. The expanded data set presented here, and future data releases, will further facilitate these cross-species studies.
